# Supplementary material for: Probing the Efficiency of 13-Pyridylalkyl Berberine Derivatives to Human Telomeric G-Quadruplexes Binding: Spectroscopic, Solid State and In Silico Analysis
Source: Int J Mol Sci. 2022 Nov 14;23(22):14061. doi: 10.3390/ijms232214061 (PMC9693123; doi:10.3390/ijms232214061)
Supplement: Supplementary file 1 [file ijms-23-14061-s001.zip › ijms-1964410-supplementary.pdf]

Article

Probing the Efficiency of 13-Pyridylalkyl Berberine Derivatives to Human Telomeric G-Quadruplexes Binding: Spectroscopic, Solid State and in Silico Analysis

Carla Bazzicalupi [1,*], Alessandro Bonardi [2], Tarita Biver [3], Marta Ferraroni [1], Francesco Papi [1,2], Matteo Savastano [1], Paolo Lombardi [4] and Paola Gratteri [2,*]

[1] Department of Chemistry, University of Florence, Via della Lastruccia 3, 50019 Firenze, Italy
[2] Laboratory of Molecular Modeling Cheminformatics & QSAR, Department NEUROFARBA—Pharmaceutical and Nutraceutical Section, University of Firenze, Via Ugo Schiff 6, 50019 Firenze, Italy
[3] Department of Chemistry and Industrial Chemistry, University of Pisa, Via G. Moruzzi 13, 56124 Pisa, Italy
[4] Naxospharma srl, Via G. Di Vittorio, 70, Novate Milanese, 20026 Milano, Italy
* Correspondence: carla.bazzicalupi@unifi.it (C.B.); paola.gratteri@unifi.it (P.G.)

Summary

Details of the Hirshfeld Surface's analysis method	page 2
Table S1	page 3
Fig S1	pages 4-5
Fig S2	page 6-7
Fig S3	page 8
Fig S4	page 9
Fig S5	page 10
Fig S6	page 11
Fig S7	page 12
Fig S8	page 13
Fig S9	page 14
Fig S10	page 15

Details of the Hirshfeld Surface's analysis method applied to the crystal structures of the adducts of ligands 2,3 and 4 with Tel12

A modified version of the Hirshfeld surface's analysis method was used to visualize the contacts established with the guanine tetrad by each ligand. Such method replaces all molecules in a crystal with a collection of spherical atoms located in the same positions (i.e. promolecules), generating a simplified version of the crystal (*i.e.* a procrystal). The Hirshfeld surface of a molecule in a crystal is defined as the region of space where the contribution of the respective promolecule to the overall procrystal electronic density, w(r), is ≥50%. Upon this mathematical object many useful contact-related properties can be mapped. Among them, popular ones include d_i, *i.e.* the point-by-point distance between the surface and the closest atom inside it, and d_e, *i.e.* the point-by-point distance between the surface and the closest atom outside it. In our analysis we used the d_{norm} parameter, which is a normalized contact distance which takes into account not only d_i and d_e values, but also the van der Waals radii (r_{vdW}) of the atoms in contact ($d_{norm} = d_{|i|}+d_{|e|}$, where $d_{|x|} = (d_x - r_{vdW})/r_{vdW}$, with x = i, e). Contacts are plotted on the surface and shown as function of colours from blue (longer than vdW contacts) to red (shorter than vdW contacts); white represents contacts equal to vdW ones.

Without giving further details, for which we refer to the specific literature (please see reference 40 in the text), we can say that the Hirshfeld surface of a specific molecule in a given crystal packing is related to its van der Waals surface, but it takes into consideration neighbouring molecules and, hence, it is useful to study the intermolecular interactions. This method is commonly applied to small molecules crystals, the overall structure of which is subjected to the calculation. Still, since the Hirshfeld surface of a given molecule is sensitive only to the direct contacts it establishes (local electronic density at any point is function of nearby molecules only), we decided to focus on the ligands and the 3′-end GGGG tetrad only, deleting remaining atoms. This prevents plotting of Hirshfeld surface as such, because in a void crystal the region where w(r) of a considered molecule is ≥ ½ becomes extremely large. We therefore used the berberine promolecule surface directly for property-plotting purposes. Promolecule usefulness and its properties have been discussed extensively during the development of Hirshfeld surface methodology (please see reference 41 in the text).

Table S1. Summary of Data Collection and Atomic Model Refinement Statistics. Values in parentheses are for the highest resolution shell.

	**2 / Tel12**	**4 / Tel12**
**Wavelength (Å)**	0.972640	0.972640
**Space group**	$P4_22_12$	$P4_22_12$
**Cell dimension (Å, deg)**	a = b = 41.68, c = 34.31<br>α = β = γ = 90.00	a = b = 42.10, c = 34.15<br>α = β = γ = 90.00
**Limiting resolution (Å)**	29.47 – 2.04	29.77 – 1.60
**Unique reflections**	2137	5303
**R_{sym} (%)**	5.6	9.6
**Multiplicity**	22.7	10.6
**Completeness overall (%)**	99.8	99.0
**<I/σ(I)>**	25.9	9.73
**CC(1/2)**	99.8	99.7
**Resolution range (Å)**	29.47 – 2.04	29.77 – 1.60
**Unique reflections, working/free**	1909 / 225	3925 / 418
**Rfactor (%)**	25.8	28.2
**Rfree(%)**	30.6	31.0
**r.m.s.d. bonds(Å)**	0.007	0.008
**r.m.s.d. angles (°)**	1.762	1.241

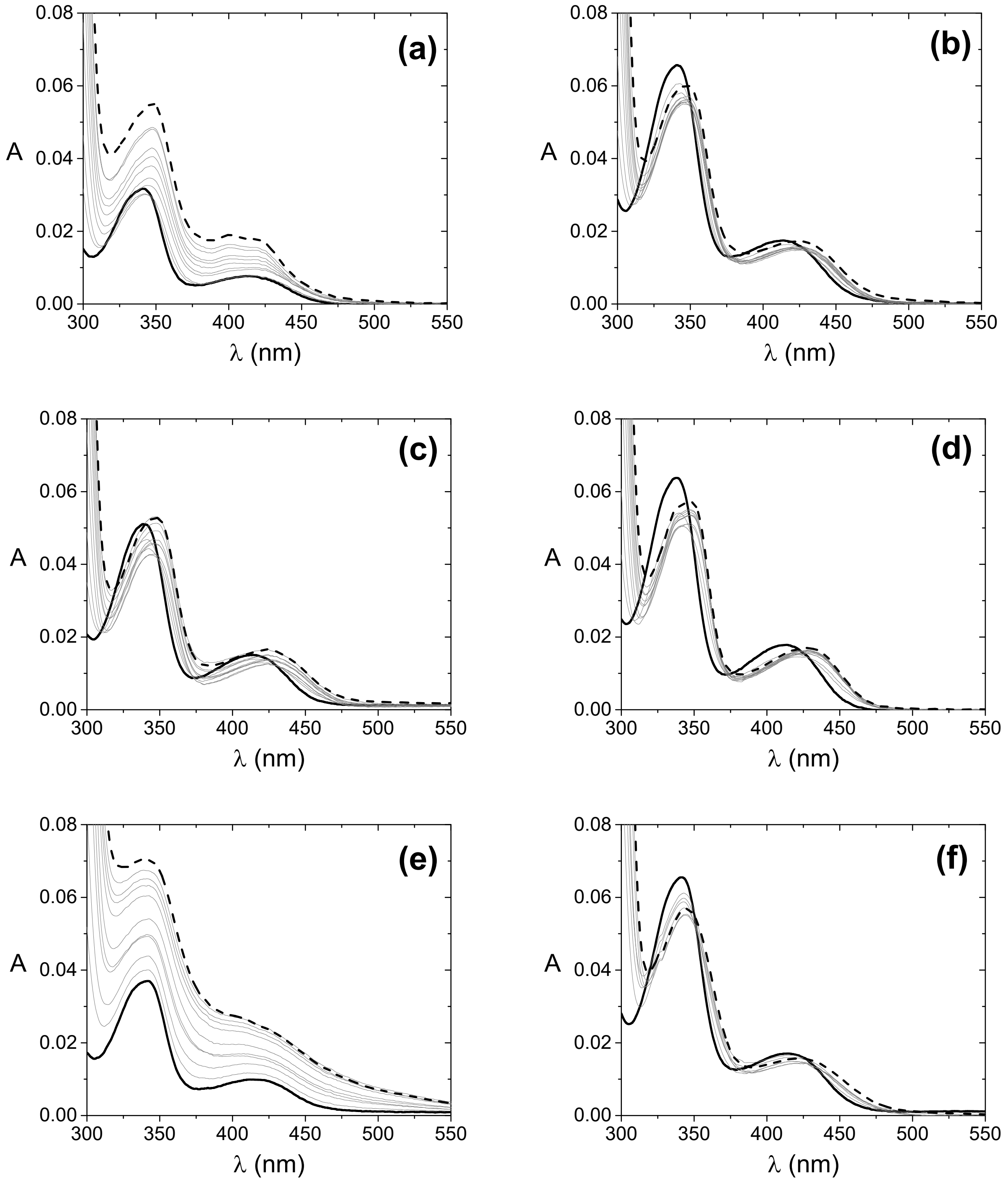

(a)
(b)
(c)
(d)
(e)
(f)
A
λ (nm)
0.00
0.02
0.04
0.06
0.08
300
350
400
450
500
550

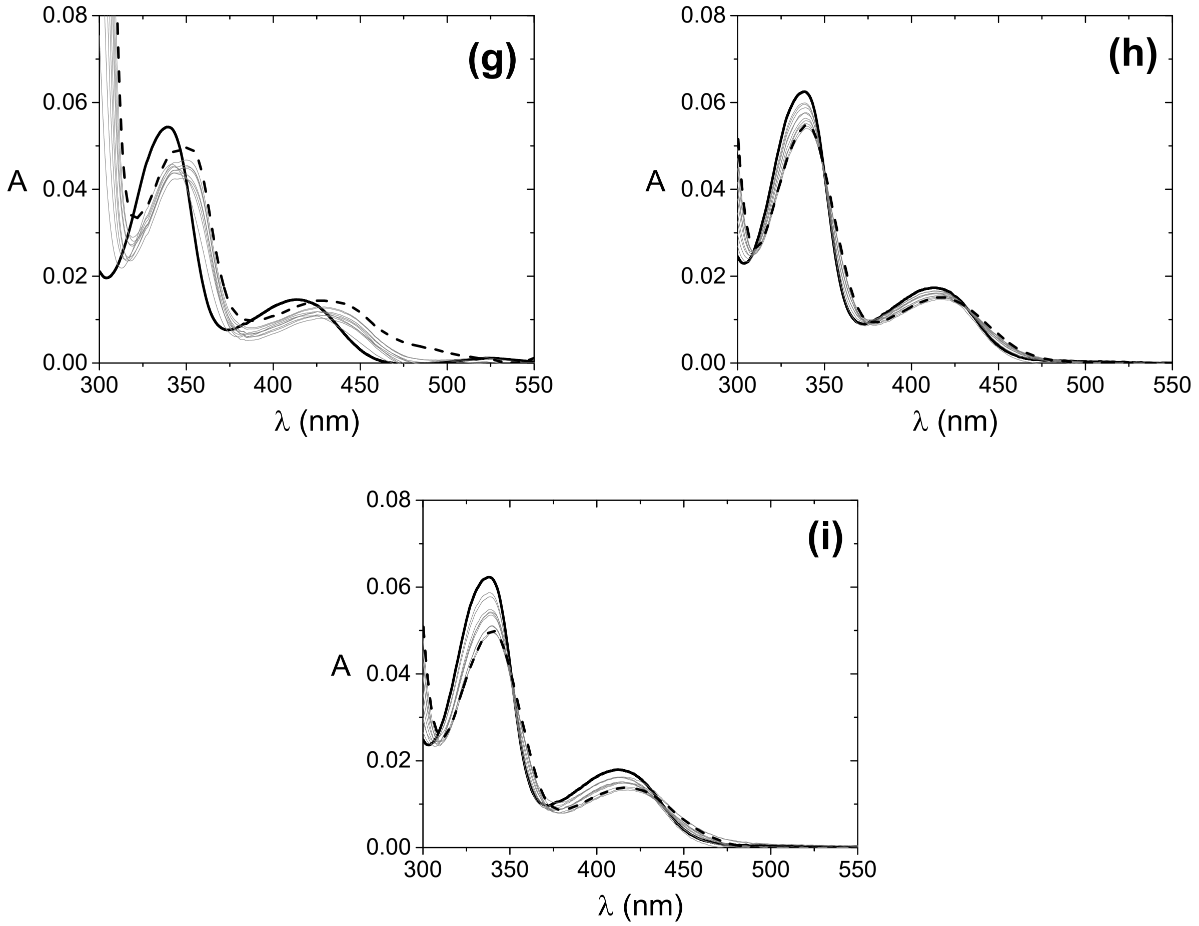

Figure S1. Examples of spectrophotometric titrations: (a) **1**/Tel12 system, [Tel12] from 0 M (full line) to 1.74×10^{-5} M (dashed line); (b) **2**/Tel12 system, [Tel12] from 0 M (full line) to 2.02×10^{-5} M (dashed line); (c) **3**/Tel12 system, [Tel12] from 0 M (full line) to 1.74×10^{-5} M (dashed line)); (d) **5**/Tel12 system, [Tel12] from 0 M (full line) to 2.02×10^{-5} M (dashed line)); (e) **1**/Tel23 system, [Tel23] from 0 M (full line) to 1.98×10^{-5} M (dashed line); (f) **2**/Tel23 system, [Tel23] from 0 M (full line) to 2.24×10^{-5} M (dashed line); (g) **3**/Tel23 system, [Tel23] from 0 M (full line) to 1.98×10^{-5} M (dashed line); (h) **4**/Tel23 system, [Tel23] from 0 M (full line) to 1.46×10^{-5} M (dashed line); (i) **5**/Tel23 system, [Tel23] from 0 M (full line) to 1.46×10^{-5} M (dashed line). In all cases [NAX] = 3.88×10^{-6} M, KCl 0.1 M, NaCacodylate 2.5 mM, pH 7.0, 25°C.

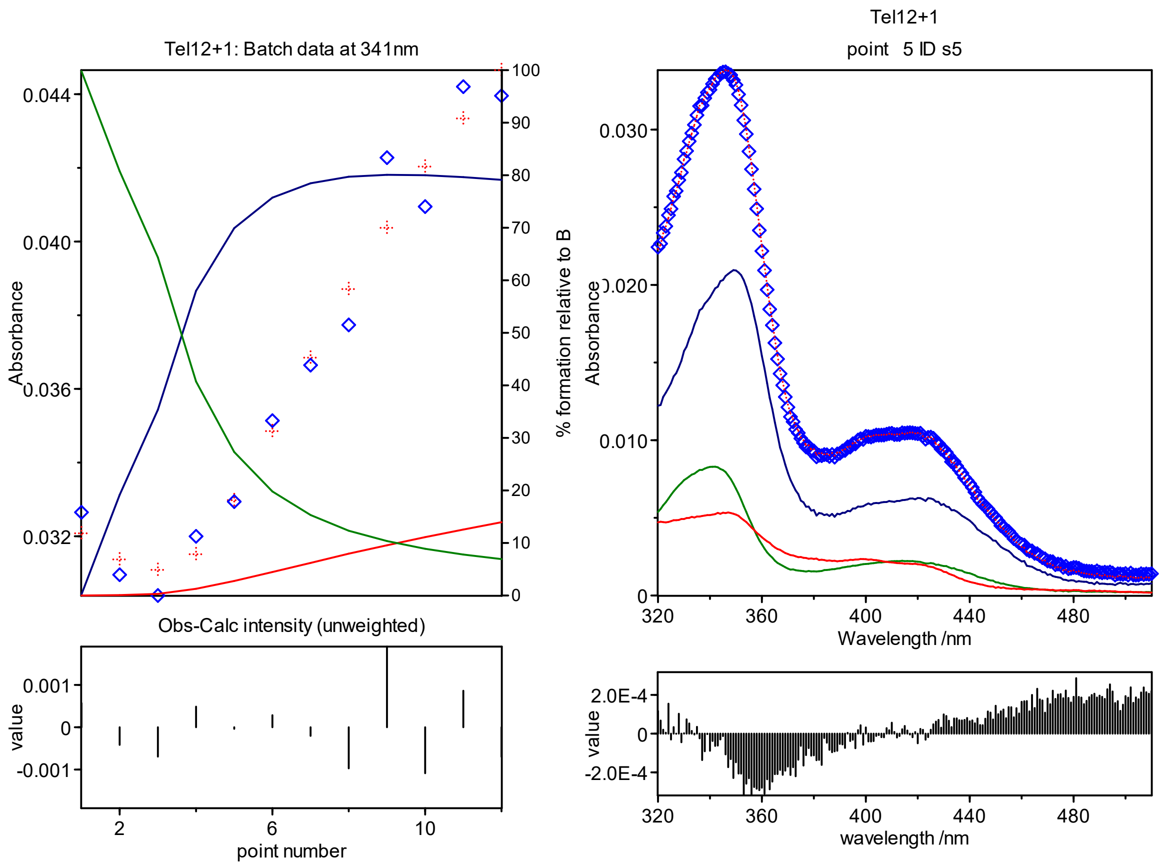

(a)

Figure S2. a). Example of HypSpec2014 analysis of the absorbance changes observed upon addition of Tel12 to **1** according to a 1:2 model A + B = AB and 2A + B = A_2B. Left: titration curve at 341 nm (open diamond = experimental, cross = calculated) and species distribution (green = B = free **1**, blue = AB = 1:1 **1**/Tel12 adduct, red = A_2B = 1:2 **1**/Tel12 adduct); % formation relative to B is the fraction of a species with respect to total berberine amount. Right: absorbance spectrum (open diamond = experimental, dashed red line = calculated) and relevant deconvolution (green = B = free **1**, blue = AB = 1:1 **1**/Tel12 adduct, red = A_2B = 1:2 **1**/Tel12 adduct). Bottom panels are the residuals, i.e. the differences (experimental value) – (calculated value). Experimental conditions: [**1**] = 3.88 × 10^{-6} M, [Tel12] from 0 to 1.74 × 10^{-5} M, KCl 0.1 M, NaCacodylate 2.5 mM, pH 7.0, 25 °C.

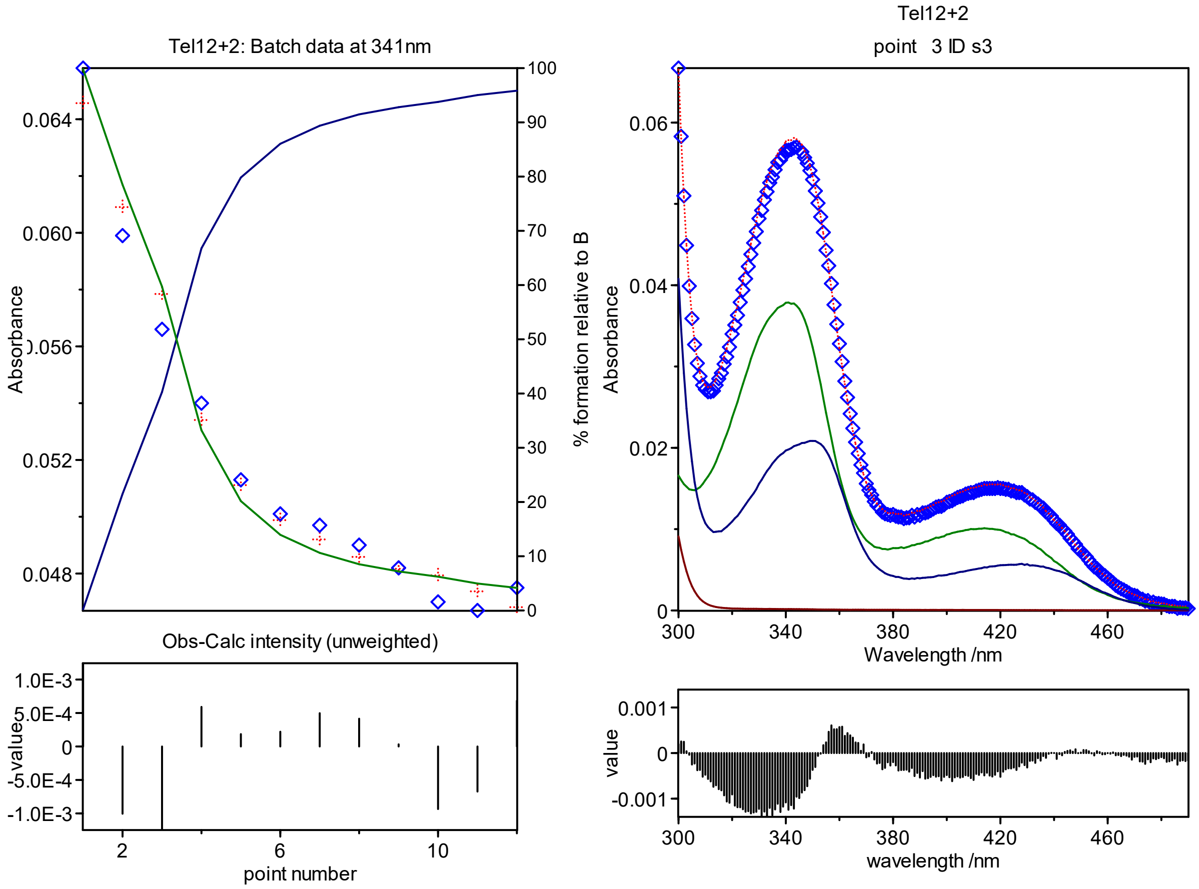

(**b**)

Figure S2. b). Example of HypSpec2014 analysis of the absorbance changes observed upon addition of Tel12 to **2** according to a 1:1 model A + B = AB. Left: titration curve at 341 nm (open diamond = experimental, cross = calculated) and species distribution (green = B = free **2**, blue = AB = 1:1 **2**/Tel12 adduct); % formation relative to B is the fraction of a species with respect to total berberine amount. Right: absorbance spectrum (open diamond = experimental, dashed red line = calculated) and relevant deconvolution (red = A = free Tel12, green = B = free **2**, blue = AB = 1:1 **2**/Tel12 adduct. Bottom panels are the residuals, i.e. the differences (experimental value) – (calculated value). Experimental conditions: [**2**] = 3.88 × 10^{-6} M, [Tel12] from 0 to 2.02 × 10^{-5} M, KCl 0.1 M, NaCacodylate 2.5 mM, pH 7.0, 25 °C.

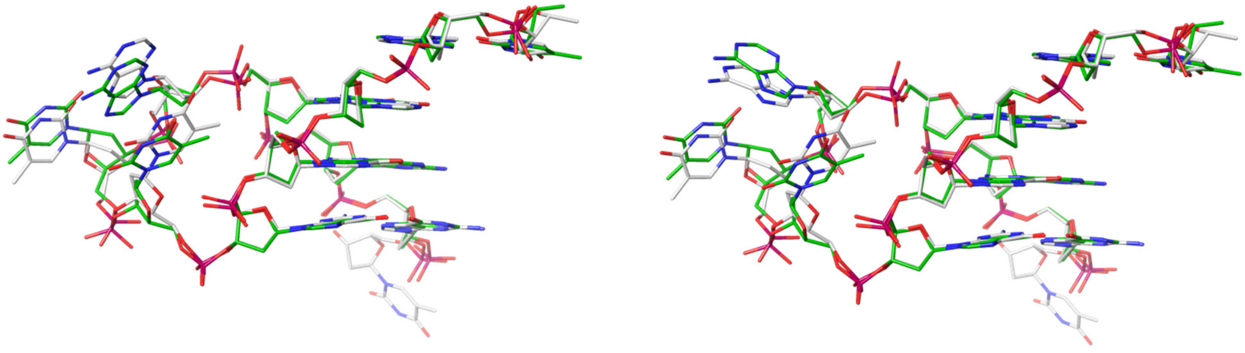

(a) (b)

Figure S3. Superposed DNA chains for the **4**/Tel12 and **2**/Tel12 structures: a) type-1 loop and b) type-3 loop (**4**/Tel12-light gray carbon atoms- and **2**/Tel12-green carbon atoms).

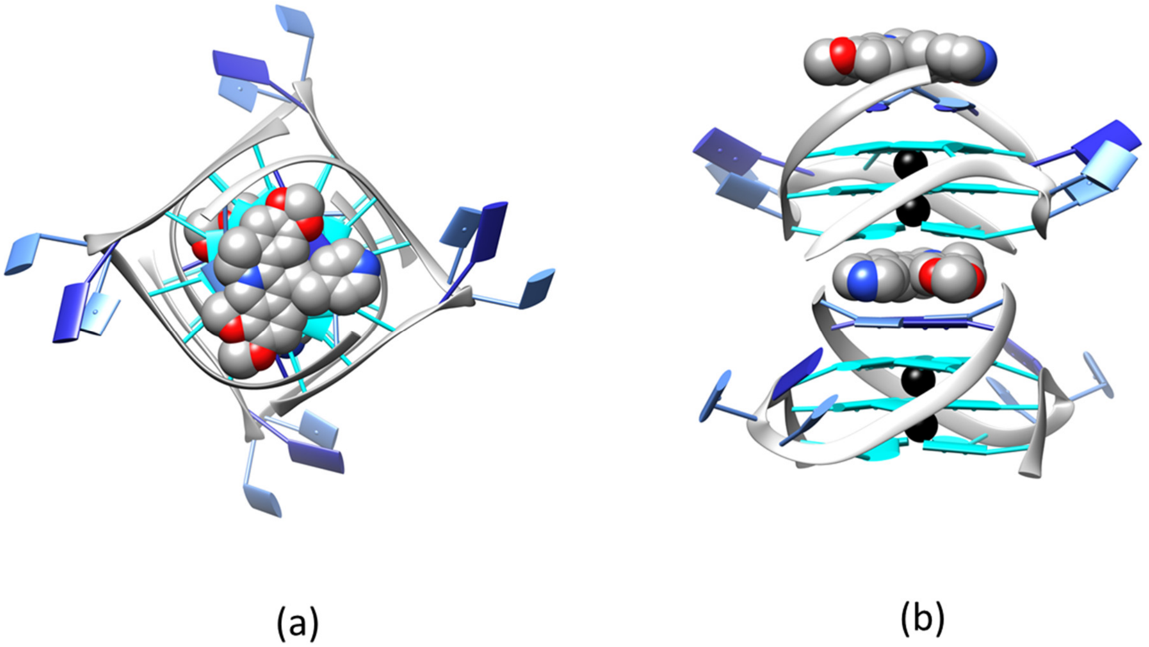

Figure S4. **2**/Tel12 crystal structure - 1:1 ligand/quadruplex adduct organized in columns growing along the c axis: (a) top view; (b) lateral view. Only one symmetry related position shown for **2** and disordered K^+ ions (color code: G = cyan, A = blue, T = light blue; potassium ions = black; ligand and water atoms = by element).

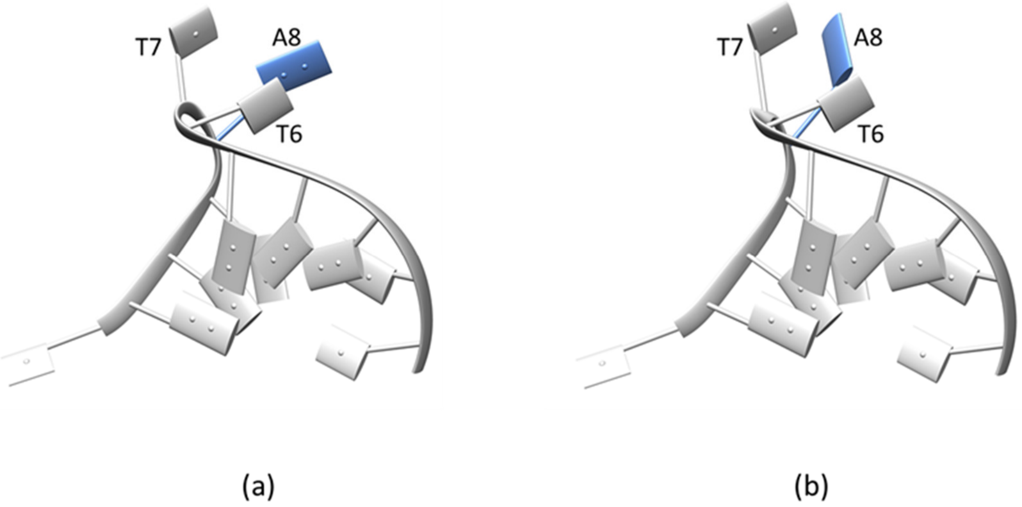

Figure S5. Type-1 (a) and Type-3 (b) loops defined by the disordered A8 (light blue) residue. For example, shown only the strand conformation in **4**/Tel12.

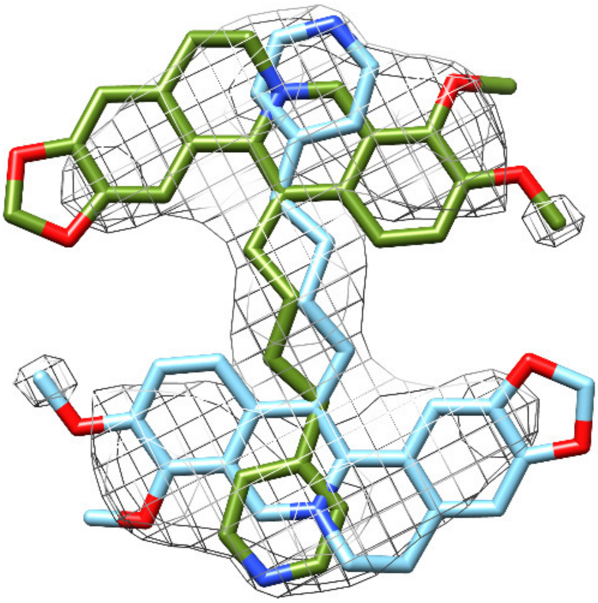

Figure S6. Crystal structure of **4**/Tel12: symmetry related ligand molecules sharing the binding site, each refined with occupancy factor 0.5. OMIT electron density map contoured at 2.5σ level.

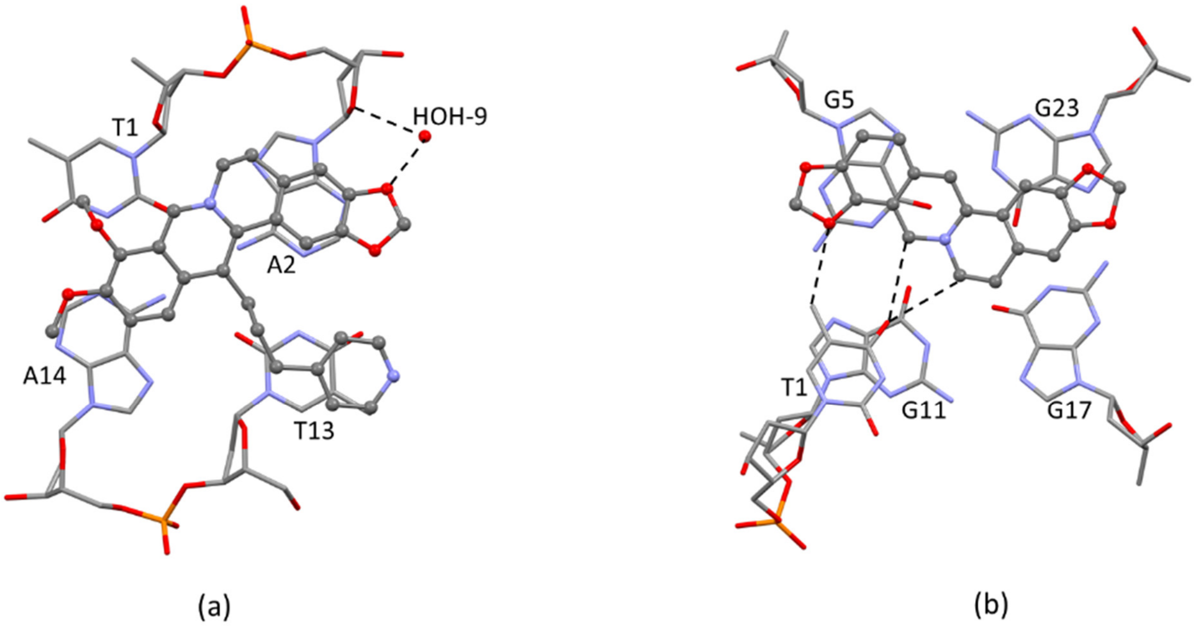

Figure S7. Water bridged and direct DNA/ligand H-bonds involving a benzodioxole oxygen in the **3**/Tel12 (a) and the Coptisine/Tel12 (b) crystal structures [35,37].

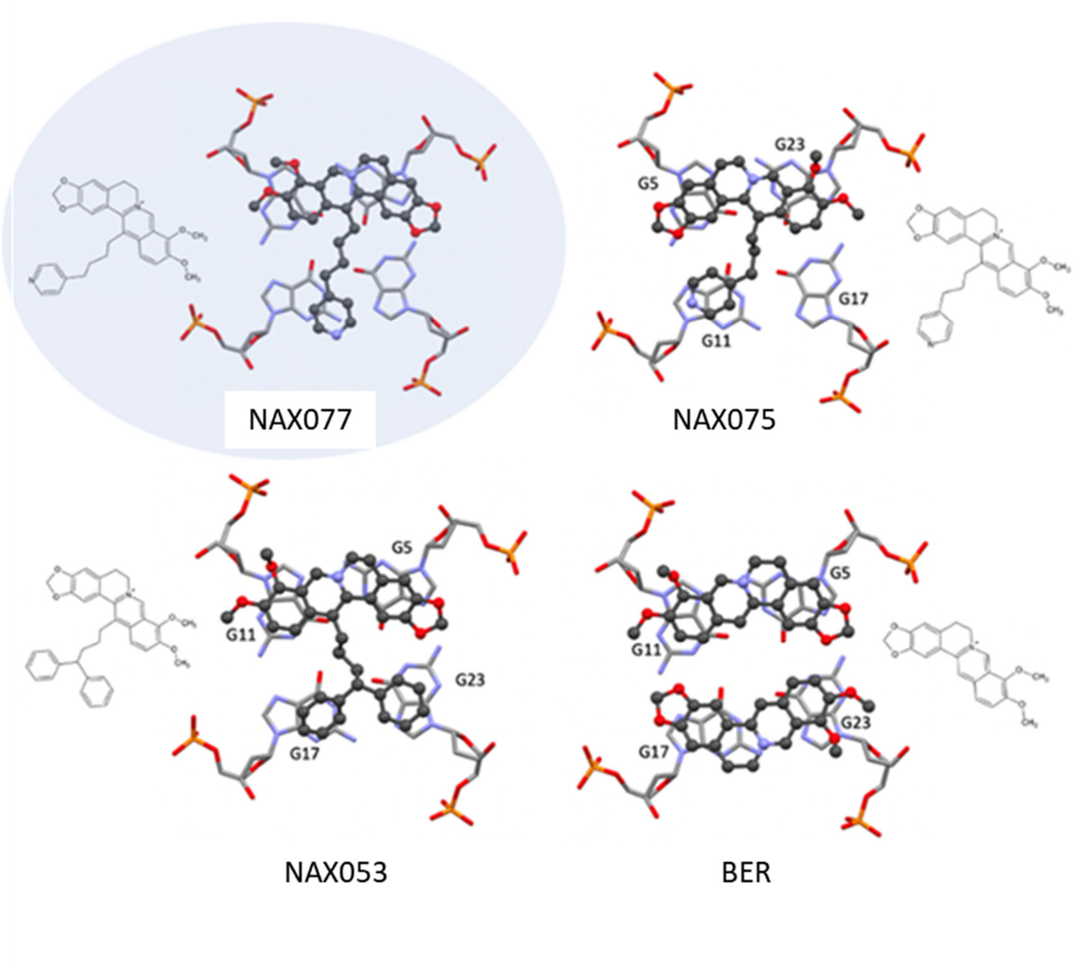

Figure S8. Comparison of the binding sites of **4** (NAX077), **3** (NAX075), NAX053 and Berberine. Ligands viewed each on top the GGGG tetrad.

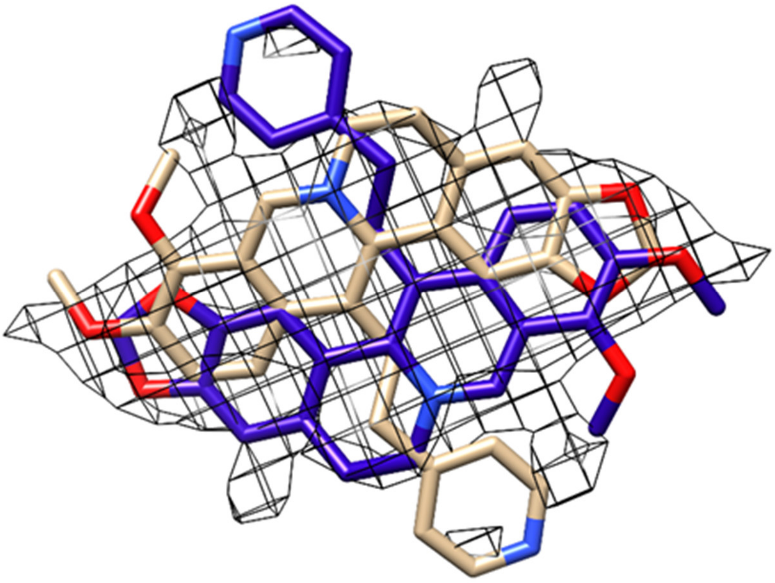

Figure S9. Crystal structure of **2**/Tel12: symmetry related ligand molecules sharing the binding site, each featuring an occupancy factor 0.5. OMIT electron density map contoured at 2σ level.

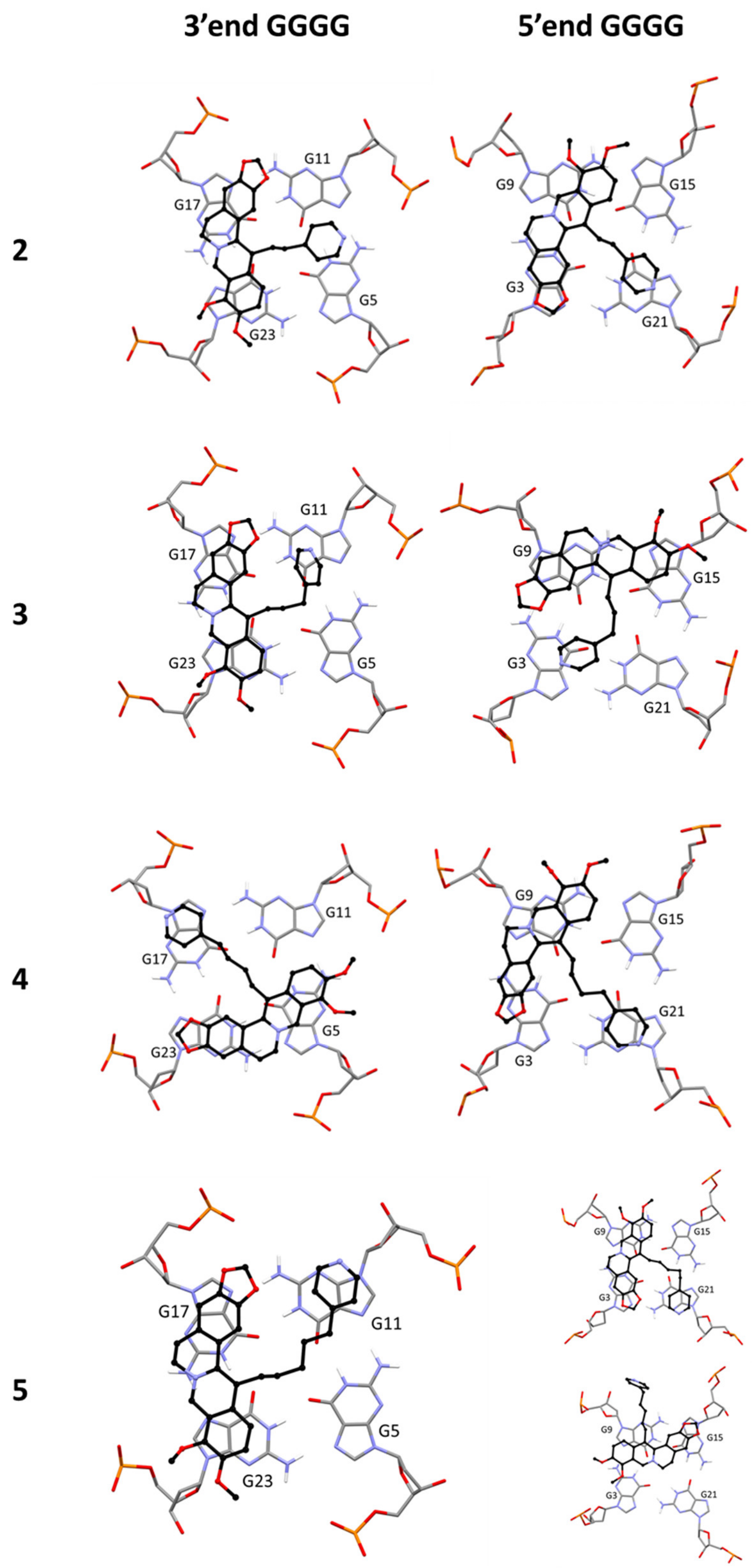

Figure S10. Molecular modelling results for the propeller telomeric G-quadruplex/NAX systems. From the top **2**, **3**, **4** and **5**, DNA residues in contact shown. For ligand **5** at the 5’end tetrad two possible orientation have been found (3’end left column, 5’end right column).
